# ASO Author Reflections: The Timing of Onset of Peritoneal Metastases from Colorectal Carcinoma Is Not an Independent Predictor of Survival Outcomes After Cytoreductive Surgery with Hyperthermic Intraperitoneal Chemotherapy

**DOI:** 10.1245/s10434-022-11842-4

**Published:** 2022-04-29

**Authors:** Michelle V. Dietz, Eva V. E. Madsen

**Affiliations:** Department of Surgical Oncology, Erasmus MC Cancer Institute, Rotterdam, The Netherlands

## Past

Cytoreductive surgery (CRS) with hyperthermic intraperitoneal chemotherapy (HIPEC) is a treatment option for patients with peritoneal metastases (PM) from colorectal carcinoma (CRC). As CRS-HIPEC is associated with considerable morbidity, it is important to select those patients who will most likely gain survival benefit from this treatment. Synchronous onset of metastases has been proposed as a negative prognostic factor for different types of colorectal metastases.^1^ Literature is inconclusive about the prognostic value of the timing of the onset of colorectal PM in patients undergoing CRS-HIPEC.^2–4^ The current retrospective study aimed to determine the impact of the timing of onset of colorectal PM (synchronous, s-PM, vs metachronous, m-PM) on survival outcomes after CRS-HIPEC.

## Present

This study shows that synchronous onset of colorectal PM was associated with poor tumor characteristics and more advanced disease.^5^ Disease-free survival (DFS) did not significantly differ between groups (median 8 vs 9 months). s-PM patients had impaired overall survival (OS) compared with m-PM patients (median 28 vs 33 months), but synchronous onset of PM was not independently associated with OS in multivariable analysis. This is probably explained by confounding factors, like tumor differentiation, lymph node status, and PCI were independently associated with OS.

## Future

To optimize patient selection for CRS-HIPEC, it is important to identify prognostic factors that could aid in preoperative patient selection. This study shows that factors such as tumor differentiation, lymph node status, and PCI are better predictors of survival outcomes than the timing of onset of PM. However, as some of these factors can only be determined during surgery, their value in preoperative patient selection is limited. Future studies should focus on the development of a prediction model of survival outcomes of patients with colorectal PM undergoing CRS-HIPEC that could be used in preoperative patient selection. The timing of onset of PM might not be an independent factor but could be useful in preoperative prediction.
